# Maternal physical activity prevents the overexpression of hypoxia-inducible factor 1-α and cardiorespiratory dysfunction in protein malnourished rats

**DOI:** 10.1038/s41598-019-50967-7

**Published:** 2019-10-08

**Authors:** Viviane O. Nogueira, Luana D. S. Andrade, Reginaldo L. Rocha-Júnior, Palloma E. D. Melo, Elisama Helvécio, Danilo A. F. Fontes, Tatiany P. Romão, Carol G. Leandro, João H. Costa-Silva

**Affiliations:** 1 Laboratory of Nutrition, Physical Activity and Phenotypic Plasticity, Federal University of Pernambuco, Vitória de Santo Antão, PE Brazil; 2 Departamento de Entomologia, Centro de Pesquisas Aggeu Magalhães - FIOCRUZ, Recife, PE Brazil

**Keywords:** Respiration, Nutrition

## Abstract

Maternal physical activity attenuates cardiorespiratory dysfunctions and transcriptional alterations presented by the carotid body (CB) of rats. Rats performed physical activity and were classified as inactive/active. During gestation and lactation, mothers received either normoprotein (NP-17% protein) or low-protein diet (LP-8% protein). In offspring, biochemical serum levels, respiratory parameters, cardiovascular parameters and the mRNA expression of hypoxia-inducible factor 1-alpha (HIF-1α), tyrosine hydroxylase (TH) and purinergic receptors were evaluate. LP-inactive pups presented lower RF from 1^st^ to 14^th^ days old, and higher RF at 30 days than did NP-inactive and NP-active pups. LP-inactive pups presented with reduced serum protein, albumin, cholesterol and triglycerides levels and an increased fasting glucose level compared to those of NP-inactive and NP-active groups. LP and LP-inactive animals showed an increase in the cardiac variability at the Low-Frequency bands, suggesting a major influence of sympathetic nervous activity. In mRNA analyses, LP-inactive animals showed increased HIF-1α expression and similar expression of TH and purinergic receptors in the CB compared to those of NP groups. All these changes observed in LP-inactive pups were reversed in the pups of active mothers (LP-active). Maternal physical activity is able to attenuate the metabolic, cardiorespiratory and HIF-1α transcription changes induced by protein malnutrition.

## Introduction

In recent years, epidemiological data from human studies and experimental evidence have verified the importance of maternal nutrition during gestation and lactation in the genesis of hypertension in adult offspring^1–5^. The potential long-term implications of undernutrition on the clinical manifestation of programmed hypertension are particularly critical because a high percentage of adults living in low-income and middle-income countries were born undernourished and have to adapt to prompt changes in postnatal diet and environment^2,6^. In addition, the association of maternal nutrition with later blood pressure is strengthened when adjusted for postnatal growth, weight, height, and body-mass-index-for-age at 2 years^2^.

In animals, a recent review showed accumulating evidence that, to a significant degree, the programmed hypertension may have a developmental origin, and the sympathetic-respiratory dysfunctions play an important role on the development of the maternal diet induced-hypertension^7,8^. Maternal protein restriction (8% casein) during gestation and lactation induced high respiratory frequency (RF) and respiratory chemosensitivity to O_2_/CO_2_^9^. The sympathetic-respiratory overactivity and amplified peripheral chemoreceptor responses were seen in juvenile rats (30 days) submitted to perinatal protein restriction^10,11^. Following the observation that sympathetic overactivity is related to the development of hypertension in organisms that suffered perinatal protein malnutrition, the molecular mechanism may include the expression of the inducible hypoxia factor (HIF-1α) in the carotid body (CB) cells^11,12^ and the expression of purinergic receptors (P2X)^13,14^. Indeed, increased HIF-1α expression was previously observed in the heart and brain of protein-restricted animals^15,16^. P2X receptors contribute to increased CB activity and its blockade may induce an antihypertensive effect^17^. Our previous study showed that early CB removal normalized RF and sympathetic nervous activity^18^. In addition, the development of the hypertension in offspring was prevented at 30 days of age in those animals submitted to perinatal protein restriction (8% casein)^18^.

Specific “re-programming” interventions, such as physical activity, may mitigate or even prevent maternal protein restriction-programmed metabolic disease^19,20^. Increasing physical activity is one of the recommendations of the World Health Organization and it is considered a broadly reachable, inexpensive, and effective intervention^21^. A meta-analysis included 13 prospective studies and showed an inverse dose–response association between levels of physical activity and risk of hypertension^22^. Maternal exercise reduces blood pressure, systemic vascular resistance, sympathetic activity, plasma renin activity, insulin resistance index, excessive body weight gain and abdominal circumference, and blood lipids^23–26^. Healthy pregnant women without medical contraindications should be encouraged to participate in regular physical activity at least 150 min per week (analogously 20–30 min per day on most or all days of the week) in moderate aerobic intensity^26^.

An experimental model of maternal physical activity has been developed to describe physiological adaptations for mother and offspring^27,28^. Pups from rats that were active before and during pregnancy (30 min/day, 5 days/week, 4 weeks) showed improved long-term memory in the object recognition task^29^. Voluntary maternal physical activity (running wheel, 30 days before breeding and during gestation/lactation) has been related to the increased somatic growth and reflex ontogeny of rat offspring during development^28^. Voluntary physical activity on the running wheel before and during gestation/lactation attenuated the effects of a maternal low-protein diet (8% protein) on the patterns of the locomotor activity of offspring rats at 60 days old^30^. However, less is known about the effects of maternal physical activity on cardiorespiratory parameters and the adaptive effects to attenuate malnutrition-induced long-term hypertension in offspring.

In the present study, we tested the hypothesis that maternal voluntary physical activity before and during gestation prevents respiratory frequency and sympathetic-respiratory overactivities and blocks the afferent inputs from the CB to brainstem acting as new insights on the re-programming of maternal diet induced-hypertension. Thus, the main goal of this study was to evaluate the effects of voluntary maternal physical activity on some parameters of cardiorespiratory and sympathetic systems. In addition, the transcriptional changes of HIF-1α, TH and P2X receptors in CB peripheral chemoreceptor were analyzed as possible molecular mechanisms.

## Results

### Maternal food intake during adaptation, pregnancy and lactation

**Figure 1:**
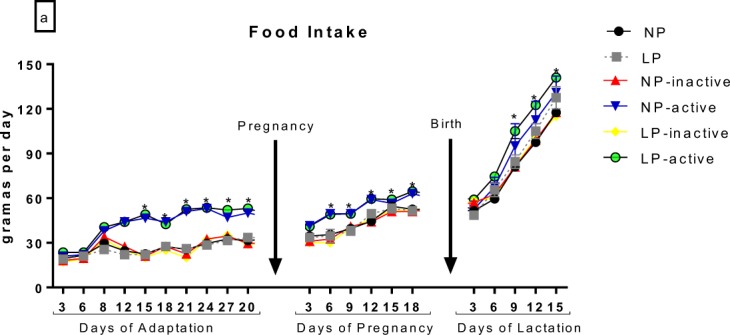
Food Intake during 30 days of adaptation, pregnancy and lactation. During adaptation and pregnancy periods, groups were constituted by NP (n = 6), LP (n = 6), NP-inactive (n = 6), NP-active (n = 6), LP-inactive (n = 6) and LP-active (n = 6). The values are presented as the mean ± S.E.M. *p < 0.05 using two-way ANOVA and Bonferroni’s post hoc test.

Descriptive data of food intake during adaptation, pregnancy and lactation are shown in Fig. 1. During these three periods, the NP-active and LP-active groups showed increased food intake compared to their respective pairs (NP, LP, NP-inactive and LP-inactive).

### Offspring’s serum biochemical parameters

The LP group showed a reduction in the serum concentration of total proteins, albumin, triglycerides and cholesterol and an increase in glucose serum concentration compared with the NP group (Table 1). Maternal physical activity did not change the biochemical parameters because the NP-active parameters were similar to those of the NP and NP-inactive groups. However, in the LP and LP-inactive groups, maternal physical activity when categorized as active was able to normalize biochemical parameters, and LP-active pups presented with the same biochemical parameters as the NP-active pups. LP-inactive pups showed no differences in terms of biochemical parameters compared to LP pups.

**Table 1:** Serum biochemical parameters (total protein, albumin, trygliceride, total cholesterol and glucose) of offspring of rats at 22 days old, from control, inactive and active dams subjected to a normo-protein diet (NP, 17% protein, n = 8) or low-proten diet (LP, 8% protein, n = 8) during pregnancy and lactation.

	22 days old					
	NP	LP	NP-inactive	NP-active	LP-inactive	LP-active
Total ptn	6.1 ± 0.1	5.3 ± 0.1*	6.1 ± 0.1	5.2 ± 0.7	4.88 ± 0.01*	5.4 ± 0.1^#^
Albumin	2.6 ± 0.1	2.1 ± 0.1*	3.1 ± 0.1	3.2 ± 0.2	2.1 ± 0.1*	2.6 ± 0.3^#^
Triglyc	130 ± 8	77 ± 6*	132 ± 7	131 ± 4	85 ± 5*	131 ± 4^#^
Total chol	122 ± 11	89 ± 6*	102 ± 4	110 ± 3	54 ± 4*	117 ± 12^#^
Glucose	107.9 ± 3.7	134.1 ± 5.3*	123.6 ± 7.1	120 ± 10.1	187.2 ± 5.1*	110.1 ± 5.2^#^

^*^Differences between LP and LP-inactive vs. NP and NP-inactive respectively (p < 0.05; two-way ANOVA). ^#^Differences between LP and LP-inactive vs. LP-active (p < 0.05; two-way ANOVA). Groups: NP-inactive (offspring of dams who received NP diet and who presented an inactive phenotype), NP-active (offspring of dams who received NP diet and who presented an active phenotype), LP-inactive (offspring of dams who received LP diet and who presented an inactive phenotype) and LP-active (offspring of dasm who received LP diet and who presented an active phenotype). Values are presented as mean ± S.E.M.

### Ventilatory parameters

**Figure 2:**
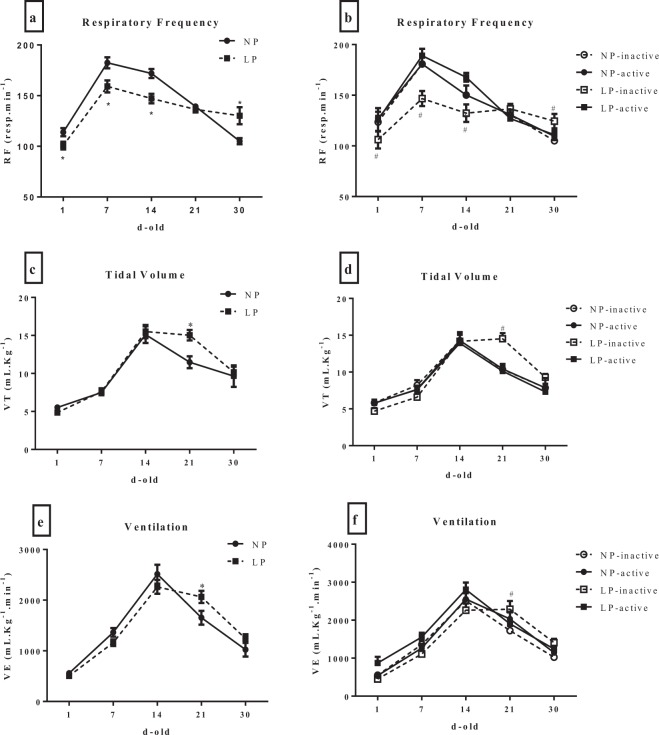
Ventilatory parameters (respiratory frequency - FR, tidal volume - VT and ventilation - VE) of offspring of rats at 1, 7, 14, 21 and 30 days old. The groups included NP (n = 8), LP (n = 8), NP-inactive (n = 8), NP-active (n = 8), LP-inactive (n = 8) and LP-active (n = 8). (**a,c,e**) Show the ventilatory parameters of rats that did not have physical activity (NP and LP groups); (**b,d,f**) show the parameters in the inactive and active groups. Values are presented as the mean ± S.E.M. *Mean values were significantly different from those of the NP group (p < 0.05; unpaired Student’s test). ^#^Mean values were significantly different after intragroup analysis (two-way ANOVA and Bonferroni’s post hoc test).

LP pups showed a reduction in the FR from the 1^st^ to 14^th^ day of life (Fig. 2a) compared with the NP pups. There were no differences when NP-active pups were compared to NP and NP-inactive pups. However, the offspring from active mothers, regardless of nutritional condition, did not present respiratory changes, as the LP-active pups showed ventilatory levels similar to those of the NP-active pups. However, LP pups showed an RF similar to those of the LP-inactive pups (Fig. 2b). Regarding VT (Fig. 2c,d) and VE (Fig. 2e,f), all groups presented the same increased values at 21 days old.

### Cardiovascular parameter

**Figure 3:**
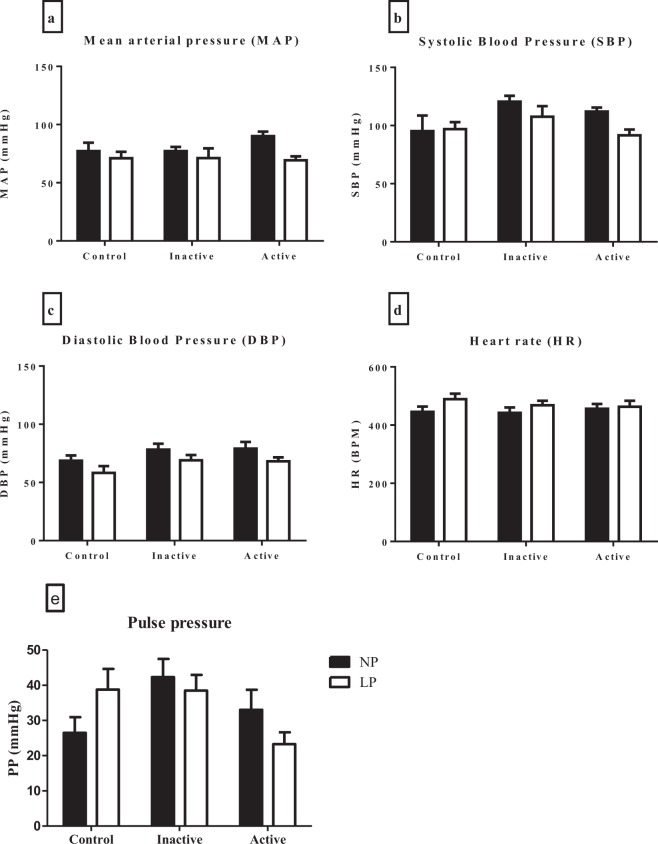
Cardiac parameters (**a**, mean arterial pressure – PAM, **b**, systolic blood pressure – SBP, **c**, diastolic blood pressure – DBP, **d**, heart rate – HR and **e**, pulse pressure) of offspring of rats, at 30 days old. The groups were constituted by NP (n = 8), LP (n = 8), NP-inactive (n = 8), NP-active (n = 8), LP-inactive (n = 8) and LP-active (n = 8). Values are presented as the mean ± S.E.M. Was used the unpaired Student’s test and for intragroup analysis used one-way ANOVA.

**Figure 4:**
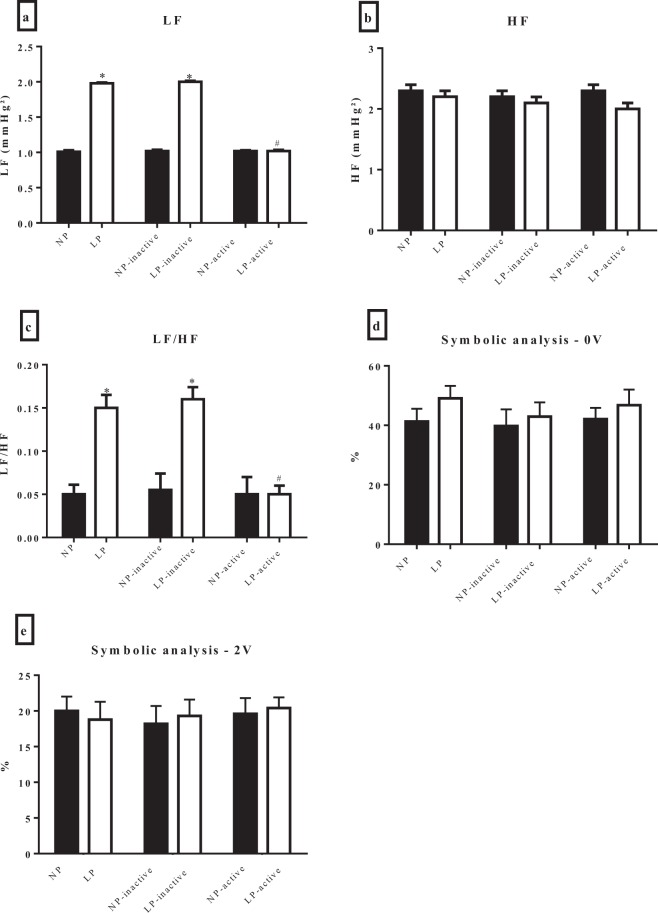
Cardiovascular variability of the systolic pressure (PS). From the pulse interval- the low-frequency bands – LF (**a**), high-frequency bands – HF (**b**), ratio LF/HF (**c**) and from the symbolic analyse- nonlinear heart rate variability of the 0 V sequences (**d**) and nonlinear heart rate variability of the 2 V sequences (**e**) of offspring of rats, at 30 days old, from mothers submitted to a normoprotein diet (NP, 17% protein, n = 8) or low-proten diet (LP, 8% protein, n = 8) during pregnancy and lactation. And from mothers that performed voluntary physical activity (NP-inativo, n = 8, NP-active, n = 8, LP-inativo, n = 8 and LP-active, n = 8). Values are presented as the mean ± S.E.M, p < 0.05. ^*^Mean values were significantly different (two-way ANOVA and Bonferroni’s post hoc test). ^#^Mean values were significantly different after intragroup analysis (two-way ANOVA and Bonferroni’s post hoc test).

There were no effects of maternal protein restriction and physical activity on hemodynamic parameters in offspring at 30 days old (Fig. 3). However, the spectral analysis of the SP in LP and LP-inactive pups showed an increase in the low-frequency oscillation - LF oscillations (Fig. 4a) when compared with the NP and NP-inactive groups, respectively. The spectral analyses of the NP-active pups were similar to those of NP and NP-inactive pups. However, the LP-active pups showed a reduction of those parameters when compared to NP-active pups. The HF components of arterial pressure showed no differences among groups (Fig. 4b). The LP and LP-inactive groups showed an increase in the LF/HF ratio (interval pulse), but LP-active pups showed similar results compared with NP-active (Fig. 4c). In the HR variability evaluated by the symbolic anlysis, the percentage of occurrences of the patterns of 0 V and 2 V sequences did not change among groups (Fig. 4d).

### qRT-PCR

**Figure 5:**
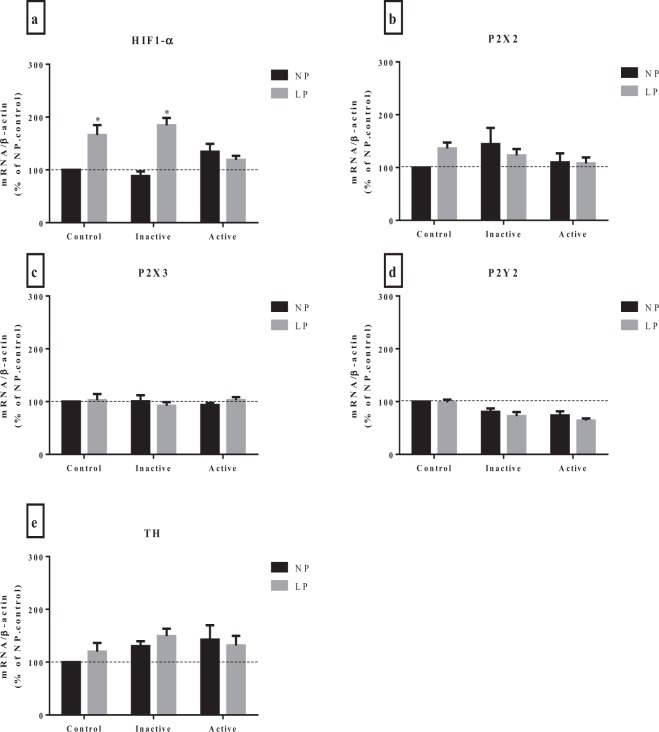
Expression of HIF-1α (**a**), P2X2 (**b**), P2X3 (**c**), P2Y2 (**d**) and TH (**e**) in CB of offspring of rats, at 30 days old, from mothers submitted to a normoprotein diet (NP, 17% protein, n = 5) or low-proten diet (LP, 8% protein, n = 5) during pregnancy and lactation. And from mothers that performed voluntary physical activity (NP-inativo, n = 5, NP-active, n = 5, LP-inativo, n = 5 and LP-active, n = 5). Values are presented as the mean ± S.E.M, p < 0.05. ^*^Mean values were significantly different after intragroup analysis (two-way ANOVA and Bonferroni’s post hoc test).

LP and LP-inactive animals showed upregulation of HIF-1α mRNA (Fig. 5a**)** compared with the NP and NP-inactive pups, respectively. The NP-active pups showed similar results to the NP and NP-inactive pups. The LP-active pups showed similar levels to those of the NP-active pups. Regarding the expression of purinergic receptors P2X2 (Fig. 5b**)**, P2X3 (Fig. 5c**)**, P2Y2 (Fig. 5d**)** and TH expressions (Fig. 5e**)**, there were no differences among groups. Neither the diet nor the level of maternal physical activity were able to alter the gene expression.

## Discussion

Pregnancy requires a significant increase in glucose-stimulated insulin secretion under conditions of normoglycaemia. However, maternal malnutrition is related to the loss of glucose sensitivity and secretory capacity in pancreatic islets, reduced serum proteins such as albumin and prealbumin, total cholesterol and indicators of lipid metabolism^31,32^. In the present study, maternal low-protein diet (8% casein) caused a reduction in the serum concentration of total proteins, albumin, triglycerides and cholesterol and an increase in glycemia. The impairment of biochemical markers of nutritional status and glucose homeostasis may lead to gestational diabetes mellitus and increased risk of chronic diseases later in life^20^. However, maternal physical activity induced a return to normal values of glucose, total proteins, albumin, triglycerides and cholesterol. Our data are aligned with previous studies on the reprograming effects of maternal physical activity in malnourished rats^19,20^. Likewise, it was observed an increased food intake-induced maternal physical activity regardless of nutritional condition. These effects are particularly important due to recovery of the loss of metabolic flexibility induced by protein restriction in skeletal muscle because, maternal metabolic dysfunction and impaired fetus growth have been associated with the development of several chronic diseases in adult life (Costa-Silva, de Brito-Alves *et al*. 2015; de Brito Alves, Toscano *et al*. 2017).

Arterial hypertension is a pathology with a multifactorial etiology, which may include renal alterations^33^, increase in the number of circulating catecholamines^34^, increase in peripheral vascular resistance^35^, sympathetic and respiratory changes^8^, among others. Respiratory function during development is an important prognostic factor for the course of neurogenic arterial hypertension and the expected lifespan of individuals who suffered perinatal undernutrition^1,7,36^. Sympathetic nerve overactivity to the cardiovascular system exhibits noticeable respiratory function-related changes which lead to rhythmic oscillations in arterial blood pressure in young and adult rats submitted to a maternal low-protein restriction^10^. In the present study, our data showed a reduction in the FR in low-protein restricted pups from the 1^st^ to 14^th^ days of age, and increased VT and VE at 21days old. Amplified respiratory modulation has been observed in multiple experimental studies of perinatal malnutrition-induced hypertension^3,9,10^. Male offspring (35 days old) of mothers with uteroplacental insufficiency, induced by bilateral uterine vessel ligation at 18 days of gestation, are also hypertensive later in life^37^. Indeed, perinatal malnourished rats display an increased sympathetic and bradycardic response to activation of peripheral chemoreceptors O_2_/CO_2_ and carotid bodies^9^. Although all respiratory parameters were not different among the groups, we observed an enhanced respiratory function in the rats of active mothers even in the protein-restricted group. The underlying mechanism can be related to increased placental insulin growth factor (IGF-1) and its receptor (IGF-1R)^38^ and vascular remodeling-induced exercise in the placenta^39^. These observations add support to the view that maternal physical exercise act positively on the respiratory function of malnourished pups, which represents an important early indication of protection to the development of hypertension.

The spectral analysis of blood pressure and heart rate variability are used to describe rhythmic and nonrhythmic fluctuations of arterial blood pressure providing indexes of autonomic cardiovascular modulation^40^. In the present study, pups of LP mothers showed an increase in the LF oscillation and LF/HF ratio, but there were no changes in HF oscilation (interval pulse). Increased LF oscilation and reduced cardiovagal influence to the heart are associated with increased risk of cardiovascular diseases, especialy hypertension and stroke^41^. Rather, we found that pups of active mothers, regardless of diet, showed apparent improvements in spectral analysis that may simply reflect the frequency-dependent nature of the baroreflex^39^. Although these results do not lead to the possible benefits of maternal physical exercise mitigating protein-restriction during the critical period of fetus-placental development, the normalization of sympathetic influence to the cardiovascular system and BP enhances the notion that physical activity *per se* enhances autonomic cardiovascular control.

The heterodimeric transcription factor HIF-1α plays an essential role in the maintenance of cellular oxygen homeostasis^42^. In response to hypoxia, stabilized HIF-1α proteins initiate the expression of genes that induce autoregulation of vascular flow in peripheral tissues, local tissue perfusion, systemic blood pressure, ventilatory rates and various responses to oxygenation controlled by the carotid body^42^. In the present study, in response to physiological levels of nutrients, LP-inactive animals showed upregulation of HIF-1α mRNA at CB. Increased expression of HIF-1α levels is associated with early response to myocardial ischemia or infarction, and the risk of arterial hypertension^15,43^. Accordingly, HIF1α mRNA increased approximately 1.3-fold in male fetal heart under maternal undernutrition (protein content in low protein food: 8.67% and in normal food: 17.9%)^16^. A recent study showed overexpression of HIF-1α in the CB of malnourished rats that were 30 days old^11^. In the study, the expression of purinergic receptors P2X2, P2X3, P2Y2 and TH expressions were not affected by maternal physical activity or diet. In contrast, the expression of the P2X2 receptor was increased by maternal undernutrition (rats deprived of protein throughout pregnancy and 42 d post-parturition) in the submucosal plexus^44^. Although the purinergic signaling at CB has been implicated in the cardiorespiratory dysfunction in some hypertensive models^17^, no changes were observed in the expression of their main receptors at CB o protein malnourished offspring. The ATP receptors are highly expressed in CB, and they also respond in an exaggerated way when ATP is applied at the site. In addition, the deletion of the ATP receptors reduced the ventilatory response to hypoxia in mice^13,45^. This finding suggests that these factors contribute to the increase in CB activity, and its blockade may provide an antihypertensive effect. However, our present study suggests no involvement of purinergic signaling in our model of maternal programming.

In addition to purinergic pathways, adrenergic neurotransmission plays an important role in the modulation of CB activity. Dopamine is the most abundant neurotransmitter in type I cells, and its release is often used as an indicator of neurosecretion, thereby promoting an increase in CB activity^46^. The carotid body and sensory and autonomic nerve fibers express tyrosine hydroxylase (TH), the rate-limiting enzyme of dopamine synthesis^47^. Some authors have considered that dopamine has an inhibitory effect, as natural stimuli such as hypoxia or acidosis are associated with increased dopamine secretion. Dopamine is a neurotransmitter capable of inhibiting carotid corpuscle activity^48,49^. Dopamine infusion is able to decrease RF. Thus, we hypothesized that the malnourished offspring would have a decreased TH expression in CB. However, this hypothesis was not confirmed by our data. In fact, we can suggest that changes in both purinergic and dopaminergic pathways at the CB are not modified by maternal physical activity and protein restriction. Nonetheless, we may establish that all these serum metabolic and cardiorespiratory changes and overexpression of HIF1α in pups from undernourished mothers were resversed in the pups of active mothers.

Thus, we conclude that maternal physical exercise plays an important role in the prevention of the early appearance of cardiorespiratory dysfunction induced by a low-protein diet during development. The effects included the recovery of the loss of metabolic flexibility, ventilatory function, and the reduction of sympathetic outflow to the cardiovascular system, as well as the normalization of hypoxia inducible factor-1α at the CB.

## Material and Methods

The experimental protocol was approved by the Ethical Commmittee of Biological Sciences Center (Protocol No. 23076.062778/2014-38), Federal University of Pernambuco, Brazil, and followed the Guidelines for the Care and Use of Laboratory Animals.

### Animals

Thirty-six 12-week-old virgin female albino Wistar rats (*Rattus norvegicus*) were obtained from the Academic Center of Vitória - CAV, Federal University of Pernambuco, Brazil, and were maintained at a room temperature of 22 ± 1 °C with a controlled light-dark cycle (dark 18.00 pm–6.00 am). Standard laboratory chow and water were given *ad libitum* during the period of adaptation (AIN-93M) and pregnancy/lactation (AIN-93G), according to the AIN-93 recommendations for periods of maintenance of laboratory rats. The AIN-93M diet was used, and during for the gestation/lactation period the AIN-93G diet was used^30,48^. During pregnancy/lactation, dams were fed either a diet 17% casein (normal protein – NP, n = 18) or 8% casein (low protein – LP, n = 18) *ad libitum*^9^. The nutritional composition of each experimental diet is shown in Table 2^30^.

**Table 2:** Composition of the diets.

Ingredients	AIN-93M* g/100 g	AIN-93G* g/100 g	
		NP	LP
Corn Starch (87% carbohydrates), g	46.47	39.74	50.34
Casein, g	14.10	20.00	9.41
Dextrinized starch (92% tetrasaccharides), g	15.50	13.20	13.20
Sucrose, g	10.00	10.00	10.00
Soya oil, g	4.00	7.00	7.00
Cellulose, g	5.00	5.00	5.00
Mineral mixture (AIN-93-MX), g	3.50	3.50	—
Mineral mixture (AIN-93-GX), g	—	—	3.50
Vitammin mix (AIN-93-VX)*, g	1.00	1.00	1.00
L-Methionine, g	0.18	0.30	0.30
Choline bitartrate (41,1% choline), g	0.25	0.25	0.25
Tert-butylhydroquinone (TBHQ), g	0.008	0.014	0.014
**Macronutrients**			
Total energy	3.44	3.56	3.56
Proteins	14%	18%	8%
Lipids	11%	18%	18%
Carbohydrates	75%	64%	74%

*^56^.

Food intake was monitored daily (24 hours) during adaptation, pregnancy and lactation. Special cages were built with a stainless steel running wheel and dams were allowed to run for a period of four weeks, as previously described^28^. After the period of adaptation (30 days), females were placed into a standard cage and mated (1 female for 1 male) for a period of 2–4 days. Females had no access to the running wheel during mating. The day in which spermatozoa were present in a vaginal smear was designated as the day of conception and day 0 of pregnancy. Pregnant rats were transferred to their original cages with free access to the running wheel throughout pregnancy, and up to postnatal day 15 to prevent the pups from running and/or being injured. On postnatal day 1, litters were reduced to 8 pups per dams, ensuring the largest possible number of males. Eventually, litters were completed to 8 pups with 2–3 females if necessary. At weaning on postnatal day 22 to avoid the influence of the estrous cycle of the females, only male pups were used. After weaning, all pups received a standard diet for rodents (52% carbohydrate, 21% protein and 4% lipids, Purina Agriband, Sao Paulo, Brazil) and water *ad libitum*. The control group, NP and LP rats with similar age and body weight were incorporated in the main study and were individually housed in a standard dimension cage without a running wheel apparatus. The litters of eight pups from each dam represent the sample and six groups were formed: NP, LP, NP – inactive (I), LP – I, NP – active (A), LP – A (Table 3). The evaluation of biochemical parameters was performed at 22 days old, ventilatory parameters were evaluated at 1, 7, 14, 21 and 30 days old; and cardiac parameters and gene expression were evaluated at 30 days old.

**Table 3:** Experimental groups classified according to daily physical activity (distance traveled, estimated calorie burned and time) in the running wheel.

Experimental groups	n	Distance traveled (km/day)	Estimated calorie burned (km/s/day)	Time (min/day)
Control	6	0	0	0
Inactive	6	≤1	≤10.0	≤20.0
Active	6	>1.0 ≤ 5.00	>10.0 ≤ 40.0	>20.0 ≤ 120.0

Adapted Santana Muniz *et al*. 2014.

### Measurements of voluntary physical activity

Female Wistar rats (initial body weight 220 g) were singly housed into an acrilic cage (cage size: 34 cm height, 27 cm width and 61 cm length)^28^. A stainess steel wheel (27 cm diameter) was placed into the cage for running physical activity with food and water *ad libitum*. A wireless ciclocomputer (Cataye, model CC-AT200W, Colorado, USA) was attached in the wheel to calculate and display running information, such trip distance (km), trip time (minutes) and estimated calorie burned (km.s^−1^.dia^−1^). Distance and time was determined by counting the number of rotations, which was translated into the number of wheel circumferences passed. Wheel circumference and diameter (measured in millimeters) were used to calibrate the cyclocomputer and then to calculate distance traveled. Calorie burned was estimated by integrating the value calculated from the speed in each second. Measurements of distance traveled, time and calories consumption were daily recorded throughout the experiment. Daily distance traveled, time and estimated calorie burned were used to classify rats in different groups according to voluntary physical activty (inactive and active) according to previous studies^28,30^.

### Serum biochemical analyses

Biochemical analyzes were performed at 22 days old. The animals were fasted for 12 hours (7:00 p.m. to 7:00 p.m.), kept in their respective cages, containing water *ad libitum* and, after this period of food deprivation, offspring were anesthetised with xylazine (10 mg/kg, ip.) and ketamine (80 mg/kg, ip.), and blood samples (~1 mL) were colleccted by plexus retro-orbital disruption. After blood collection, the samples were placed in tubes without anticoagulants and then centrifugation was performed at 3500 *g* for 10 minutes to obtain the serum. The supernatant was removed, transferred to an Eppendorf tube and stored at −20 °C, which was then sent to perform the biochemical analyzes of total protein, albumin, triglyceride, total cholesterol and fasting glucose using respective commercial kits and following recommendations and procedures determined by the manufacturer company (Labtest Diagnostica, MG, Brazil)^9^.

### Respiratory recordings and analyzes

Respiratory frequency (RF), tidal volume (VT) and ventilation (VE) were performed using the whole body plethysmography method^50^. According the rats’ age were placed into a plexiglas chamber of 212 mL (1 and 7 postnatal day), 700 mL (14 postnatal day) or 5 L (21 and 30 postnatal day). The animals were maintened for a period of acclimatization (~10 a 60 min) and the chamber was flushed with humidified air and maintained at 25 °C. After the rats had been acclimatized, their ventilatory parameters were recorded as the airflow to the chamber was suspended for short periods (~2 min), and the pressure oscillations caused by breathing were captured by a pressure differential transducer connected to a signal amplifier (ML141 Spirometer, PowerLab; ADInstruments^®^). The signals were then captured by an acquisition system and data analysis was performed (PowerLab; ADInstruments^®^). All of the data were analyzed off-line with the use of appropriate software (LabChart 7 Pro; ADInstruments^®^). Tidal volume (VT) and respiratory frequency (RF) were calculated as described by Malan (1973)^50^, and ventilation (VE) was obtained as the product of VT and RF. These parameters were calculated using a period of 10 s of respiratory recordings in conscious rats when they were quiet and presenting no body movements. The data recorded when the rats were moving inside the chamber were excluded from analysis because the respiratory activity was contamin ated by larger oscillations in the pressure inside the chamber. The rats were weighed on the day of recordings to determine the body weight, which was used to correct the ventilation index for each animal^9,51^.

### Cardiovascular recordings

Thirty postnatal day, the animals were anesthetized with ketamine (80 mg.kg^−1^) and xylazine (10 mg.kg^−1^), and the femoral artery PE-50 was connected to PE-10 (Clay Adams, Parsippany, NJ, USA). The catheter were filled with heparinized saline (NaCl 0.9% + heparine 0.05%), tunnelled subcutaneously and exteriorized through the back of the neck. After surgery, the animals received injection of ketoprofen (5 mg.kg^−1^ i.p.) and a period of 18–24 hours (sufficient recovery time for this surgical procedure, as previously described by Nogueira (2018). Mean arterial pressure (MAP), systolic blood pressure (SBP), dyastolic blood pressure (DBP), heart rate (HR) and pulse pressure (diference between SBP and DBP) were recorded in unanesthetized freely-moving animals by connecting the arterial catheter to a pressure transducer. The signals were amplified (ML866/P, ADInstruments^®^, Power Lab, Bella Vista, NSW, Australia), sampled at 2 kHz, digitalized (Power Lab, model 4/30, ADInstruments^®^) and recorded using appropriate software (LabChart7 Pro, ADInstruments^®^)^51^. Recordings of baseline pulsatile arterial pressure (PAP), MAP and HR were performed for 60 min. The cardiovascular autonomic evaluation was perfomed using the frequency domain analysis of the systolic arterial pressure (SAP) by an appropriate software program (version 2.4, available at https://www.sites.google.com/site/cardioseries/home)^10^. The power of the oscillatory components obtained from rats of the NP and LP groups was quantified into two frequency bands: low-frequency (LF: 0.20–0.75 Hz), representative of the modulatory effects of sympathetic activity controlling vascular tonus and heart activity; and high frequency (HF: 0.75–3.0 Hz), associated to a respiratory or parasympathetic modulation of heart rate and arterial pressure^52,53^. To assess the sympathovagal index to the heart, the LF/HF ratio of the IP variability was calculed. Moreover, the symbolic analysis was used, a non-linear method based on the conversion of the series into a sequence of symbols^54,55^.

### Sample for qRT-PCR

A group of offspring (n = 30) at 30 days old, free of any experimental procedure, was separated for analysis of gene expression. The animals fasted for 6 hours and were then euthanized. Immediately after an incision was made at the height of the trachea, in the isolate carotid artery and sectioned at its bifurcation, with the intention of collecting the CB. The sample was frozen rapidly and stored at −80 °C.

### Quantitation of genes transcribed by qRT-PCR

The expression levels of HIF-1α, P2X2, P2X3, P2Y2 and TH were evaluated by real-time quantitative Reverse Transcription PCR (qRT-PCR). Total RNA was extracted from CB tissues with a miRNeasy Mini kit (Qiagen^®^, Hilden, Germany) according to the manufacturer’s instructions. We used RNA extraction with TRIzol™ (Invitrogen, Van Allen Way Carlsbad, CA 92008, EUA), according to the manufacturer’s instructions. Samples were treated with DNase™ (USB) and quantified in a NanoDrop 2000c spectrophotometer (Thermo Scientific, Wilmington, Delaware, EUA). The primer sequences used in this study are reported in Table 4. Reactions were performed in a 96-well plate (20 µL reaction per well) using a QuantiTect SYBR Green RT-PCR kit (Qiagen^®^, Hilden, Germany) for one-step quantitative RT-PCR, according to the manufacturer’s instructions. Each RNA sample was normalized to 100 ng, and reactions were prepared containing SYBR green master mix, reverse transcriptase, forward/reverse primers (0.2 µM each) for the target and the endogenous control genes (0.1 µM each). qRT-PCR for each sample was performed in duplicate. Reactions were incubated at 50 °C for 30 min to allow reverse transcription; 95 °C for 15 min, to activate Taq; and 40 cycles of denaturation (94 °C for 15 s), annealing (60 °C for 30 s) and elongation (72 °C for 30 s). qPCR results from each gene (including the housekeeping gene) were performed using an ABI 7500 real-time PCR system (Applied Biosystems^®^, Foster City, California, EUA). Relative quantification analyses were performed by the 2^-ΔΔCT^ method and derived from a reference sample (LIVAK; SCHMITTGEN, 2001). Gene expression data were normalized using β-actin as the housekeeping gene, using the 7500 software version 2.0.4 (Applied Biosystems^®^).

**Table 4:** Sequences of primers used for the real-time RT-PCR analysis.

Gene	F/R	Sequencia 5′-3′	Tm (°C)
β-actin	F	CCTGACCCTGAAGTACCCCATTG	60
	R	CATGGCTGGGGTGTTGAAGGTC	
HIF-1α	F	GCGAGAACGAGAAGAAAAATAGG	60
	R	GCACCTAGAAGTTTCCTCACACG	
P2X2	F	TGGGACTACGAGACGCCTAA	60
	R	CAGGATGAGAAGCTGCACCA	
P2X3	F	TAAAGGGACAGGCTCCCCAT	60
	R	CCTACAGGACAGGGAGACGA	
P2Y2	F	AAAGAGGAACGAACACCGGG	60
	R	GTCACGTAATGGGCTCTCCC	
TH	F	AAAGAGGAACGAACACCGGG	60
	R	GTCACGTAATGGGCTCTCCC	

F: forward sequence; R: reverse sequence.

Sequences of Inducible Hypoxia Factor 1α (HIF-1α), Purinergic Receptor P2X 2 (P2X2), Purinergic Receptor P2X 3 (P2X3), Purinergic Receptor P2Y2 (P2Y2), Tyrosine Hydroxylase (TH).

### Statistical analyses

Measurements of biochemical, ventilation, blood pressure and gene expression are presented as means ± S.E.M. The NP and LP group were compared using the unpaired Student t-test, the effects of physical activity were compared using the ANOVA two-way test, followed by the Bonferroni post hoc. Differences between groups with p values of <0.05 were considered significant. GraphPad Prism 5.0^®^ software was used to perform the analysis.
